# Intelligent Corrosion Diagnosis of High-Strength Bolts Based on Multi-Modal Feature Fusion and APO-XGBoost

**DOI:** 10.3390/s26082520

**Published:** 2026-04-19

**Authors:** Hanyue Zhang, Yin Wu, Bo Sun, Yanyi Liu, Wenbo Liu

**Affiliations:** 1 College of Information Science and Technology & Artificial Intelligence, Nanjing Forestry University, Nanjing 210037, China; 2 College of Automation, Nanjing University of Aeronautics and Astronautics, Nanjing 211106, China

**Keywords:** acoustic emission, corrosion diagnosis, multimodal learning, wireless sensor networks, nondestructive testing

## Abstract

High-strength bolts are critical structural components that are highly susceptible to corrosion in complex environments, posing significant threats to structural safety and reliability. Although acoustic emission (AE) technology has been widely applied in structural health monitoring, existing studies mainly focus on damage mode identification or source localization, while the identification of corrosion evolution stages based on AE signals remains insufficient. This study develops an intelligent corrosion diagnosis framework for high-strength bolts by integrating multimodal feature fusion and optimized machine learning. AE signals are first collected from the near-end and far-end of bolts using a wireless sensor network and then transformed into time–frequency representations via continuous wavelet transform (CWT). The resulting time–frequency images are fed into a modified ResNet-18 network to extract deep features, while statistical features are simultaneously extracted from the raw signals to preserve global information. These heterogeneous features are subsequently fused to form a comprehensive representation of corrosion characteristics. Furthermore, an artificial protozoa optimizer (APO) is introduced to adaptively optimize the hyperparameters of the XGBoost model. The results demonstrate that AE signals generated by hammering bolts with different corrosion levels can be successfully distinguished. The proposed method achieves high accuracy in corrosion stage classification and outperforms conventional approaches. Even when evaluated on an additional M30 bolt dataset, the proposed method maintains robust performance, demonstrating excellent generalization capability across different bolt sizes. These results demonstrate the practical potential of the proposed method for intelligent bolt corrosion diagnosis.

## 1. Introduction

**Figure 1:**
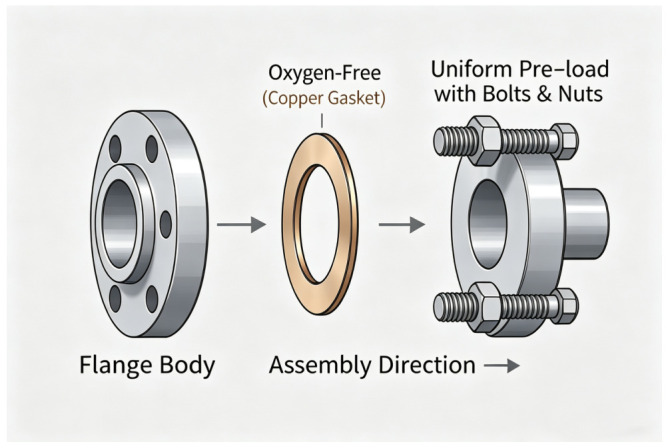
Typical structure of a bolted flange connection.

High-strength bolts are indispensable connecting components in modern engineering structures. Their service status directly affects the overall safety and operational stability of key infrastructure such as bridges, wind turbines, pressure vessels and large machinery [1,2]. Among various connection forms, bolted flange joints are widely used in engineering practice. A typical bolted flange joint consists of flanges, bolts, nuts, and sealing gaskets, as shown in Figure 1. Bolt preload generates the required clamping force between the flange faces, thereby achieving both reliable structural connection and sealing performance.

However, when exposed to harsh environments such as moisture, salt spray, or industrial pollution for a long time, bolts will inevitably suffer from electrochemical corrosion [3]. Corrosion degrades the load-bearing capacity of bolts and induces local stress concentration due to the volumetric expansion of corrosion products. Over time, this may lead to loose connections, fatigue fractures, and even serious structural safety accidents [4,5]. In order to achieve predictive maintenance and reduce major hidden dangers, it is particularly necessary to develop intelligent monitoring technology for bolt corrosion with early warning and accurate assessment functions.

From a mechanistic perspective, mechanical stress has a significant modulation effect on corrosion behavior: residual tensile stress or preload can accelerate the evolution of pits into cracks and change the damage incubation and expansion process through mechanisms such as corrosion fatigue, stress corrosion cracking, and hydrogen-induced cracking [6,7,8,9]. Therefore, the stress and preload measurement of in-service bolts has long been a focus of attention. Common methods include multi-axis force measurement, fiber Bragg grating, etc. [10,11]. In addition, distributed fiber optic sensing (DFOS) has also been applied to corrosion monitoring because of its capability for continuous spatial measurement [12]. Representative studies include Ahn et al. [13], who revealed the attenuation law of clamping force caused by local bolt corrosion through artificial damage tests and proposed a method for estimating the re-clamping cycle or replacement timing. Shah et al. [14] used guided-wave ultrasonic testing to investigate the relationship between bolt head corrosion damage and preload loss, and found that although the energy transmission trend was unstable, the waveform characteristics showed significant differences. Zhang et al. [15] proposed an “intelligent cloud bolt” integrating a multifunctional sensing layer, an ultrasonic force measurement module, and a 4G communication module for remote monitoring. Through intelligent monitoring, it can achieve accurate monitoring of preload and remote data transmission. However, these methods mostly focus on mechanical quantity modeling or active stress perception, and have not yet solved the problem of real-time diagnosis and classification of bolt corrosion levels in service environments. This shortcoming still restricts the comprehensive evaluation of service safety and remote intelligent operation and maintenance.

In recent years, researchers have widely adopted intelligent algorithms to identify industrial corrosion damage at different stages. Sheikh et al. [16] extracted the statistical features of AE signals in the accelerated corrosion experiment of low-carbon steel and used a multi-class machine learning model to achieve high-precision classification of five corrosion severity levels. Similarly, Pan et al. [17] employed acoustic emission techniques combined with Gaussian mixture model clustering to achieve damage pattern recognition in corroded beams, successfully distinguishing different damage modes and their evolution characteristics. Han et al. [18], based on a multi-scale convolutional neural network (MS-CNN), combined with the multimodal propagation and dispersion effect of ultrasonic guided waves, obtained the anchor guided wave waveform through electrochemical corrosion experiments, and achieved accurate diagnosis of the degree of anchor corrosion. Li et al. [19] proposed a percussion acoustic model TriNet for high-strength bolts in wind farms, which integrates convolutional neural networks, attention mechanisms and routing algorithms and introduces confidence weighting. Kralovec’s AICorrSens project in the aviation field integrates ultrasonic, electrochemical and environmental sensors and combines AI to achieve real-time detection, location, quantification and prediction of corrosion status [20]. In addition, Zhou et al. [21] applied a deep residual network to time–frequency representations of acoustic emission signals, achieving accurate source localization in complex structures. These studies demonstrate that data-driven methods, ranging from traditional machine learning to deep learning, have significantly improved the capability of corrosion-related damage identification, particularly in extracting discriminative features from complex acoustic emission signals.

Acoustic emission (AE) is a dynamic nondestructive testing technique capable of capturing elastic waves generated by material damage, and has been widely used for monitoring crack propagation and corrosion processes [22,23,24]. Compared with conventional methods such as ultrasonic and radiographic testing [25], AE enables real-time detection and is highly sensitive to early-stage micro-damage [23]. In addition, AE monitoring is less constrained by structural accessibility and is suitable for in-situ monitoring of bolt corrosion [26]. It has advantages over magnetic particle [27] and magnetic memory testing [28]. AE technology can also achieve real-time online monitoring that cannot be achieved by piezoelectric testing [29] and tapping testing. For example, Tian [30] used acoustic emission technology to monitor the fracture of high-strength bolts and proposed a dimensionless index using signal processing methods such as wavelet packet decomposition to identify the acoustic emission source of high-strength bolt damage and provide early warning of fracture failure. Gao et al. [31] used continuous wavelet transform to extract time–frequency features of AE signals, and combined with a deep learning model to achieve high-precision identification of the degree of bolt looseness. Li et al. [32] employed an optimized XGBoost-based model for acoustic emission signal analysis under complex propagation conditions, achieving reliable identification performance. Therefore, this study adopts a modified ResNet-18 network for deep feature extraction from time–frequency representations and employs XGBoost as the core classifier, improving the accuracy and robustness of bolt corrosion grade classification.

From a data-driven perspective, recent studies have further applied deep learning models for corrosion-related fault classification. In investigating equipment failures caused by industrial corrosion, current research mostly combines deep learning models to achieve fault classification. Sheikh et al. [16] conducted accelerated corrosion tests on low-carbon steel samples, collected AE signals at different corrosion stages, combined AE mean, root mean square, energy and kurtosis features, and used naive Bayes, BP neural network and radial basis function neural network (RBF-NN) to achieve high-precision classification of five levels of corrosion severity. Han et al. [18], based on a multi-scale convolutional neural network, achieved accurate diagnosis of anchor corrosion. These methods include Fourier transforms, wavelet analysis, and the application of deep learning algorithms, which have significantly improved the depth and accuracy of sensor acoustic emission analysis [33].

However, most studies still rely on single-point or single-channel signals, making it difficult to explore spatial correlations across locations. To improve robustness and early warning capabilities, researchers are currently exploring multi-sensor collaboration and multimodal fusion. Existing studies such as Li et al. [34] proposed a multi-node AE monitoring method based on wireless sensor networks, which achieved real-time online assessment of structural health status. Wang et al. [35] combined acoustic emission and electrochemical noise sensors for collaborative monitoring to achieve information fusion and multi-sensor complementary identification. Haitz et al. [36] achieved five-dimensional feature fusion classification for industrial corrosion detection by constructing a fusion laser depth and AE multi-channel feature space. Chernov et al. [37] fused RGB and AE signals for steel pipe weld defect detection, further verifying the application potential of heterogeneous multi-sensor fusion in structural health monitoring.

**Figure 2:**
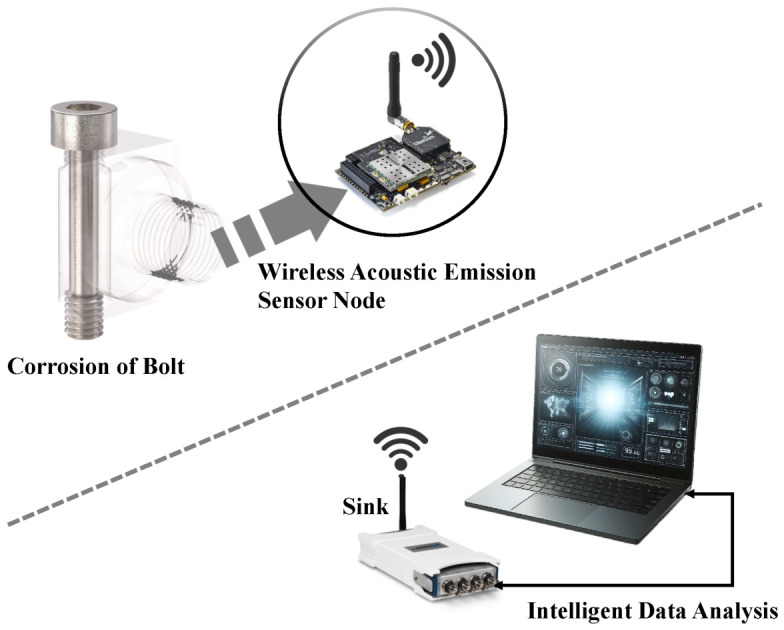
Overall system structure framework diagram.

In general, existing research has made progress in mechanical consequence modeling or single-point AE identification, but still lacks a distributed, multi-node collaborative and intelligent fusion framework for engineering environments. To differentiate the proposed approach from existing multimodal AE-based diagnosis frameworks, this work develops a distributed corrosion monitoring framework that combines a multi-node AE sensing system based on STM32 and LoRa with a two-stage feature fusion strategy and a machine-learning classifier. The overall system structure is shown in Figure 2. The main contributions are as follows:

- An intelligent corrosion diagnosis framework for high-strength bolts is developed by integrating multimodal feature fusion with optimized machine learning, enabling effective identification of corrosion stages based on acoustic emission signals.
- A dual-source feature representation is constructed by combining deep features extracted from CWT time–frequency images with statistical features derived from raw AE signals, providing a comprehensive characterization of corrosion-related information.
- An artificial protozoa optimizer (APO) is introduced to adaptively optimize the hyperparameters of the XGBoost classifier, thereby improving classification performance.
- A wireless acoustic emission monitoring and intelligent analysis system based on STM32 and LoRa is developed, enabling an integrated workflow of distributed data acquisition and corrosion stage diagnosis, and providing support for engineering applications.

## 2. Methodology

To achieve real-time diagnosis of bolt corrosion, this study employed acoustic emission technology, combined multimodal feature fusion with machine learning methods to improve identification accuracy, and constructed a multi-node AE sensor network based on STM32 and LoRa for distributed monitoring. Five corrosion levels were simulated through electrochemical acceleration experiments. The near- and far-end sensor signals were converted into continuous wavelet transform time–frequency plots, and the dual channels were input into a modified ResNet-18 network for deep feature extraction. The 512-dimensional deep features and statistical features were then combined into a 524-dimensional feature vector. Finally, an XGBoost classifier optimized by the APO algorithm was used to determine the corrosion level.

### 2.1. Hardware and Data Acquisition System

**Figure 3:**
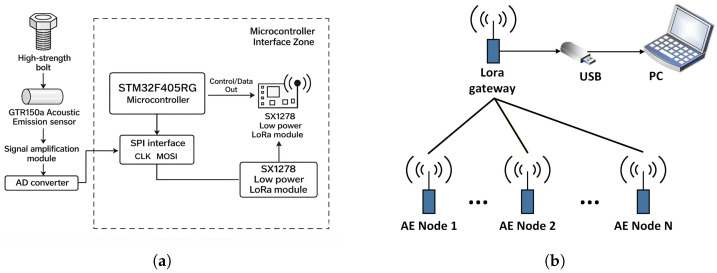
(**a**) Overall architecture of the wireless acoustic emission system; (**b**) multi-node wireless networking topology. (Ellipses indicate omitted AE nodes).

This paper develops a multi-node wireless acoustic emission sensor network (WAESN) system based on the STM32F405RG microcontroller and LoRa wireless communication. The proposed system is designed for real-time, low-power monitoring and corrosion grade identification of high-strength bolts. It mainly consists of five parts: AE acquisition nodes, an embedded control unit, a wireless communication module, a central receiving gateway, and a host computer platform. Figure 3 shows the overall architecture of the wireless acoustic emission system and the multi-node networking topology. The system supports multi-channel AE signal acquisition, long-distance wireless transmission, and remote visualization and analysis of corrosion status.

The acoustic emission (AE) signals of bolts in a normal state are mainly distributed below 125 kHz, whereas corrosion-induced deformation typically generates AE signals in the range of 125–150 kHz. Based on this frequency characteristic, a GTR150a AE sensor (Hunan Endity Technology Co., Ltd., Changsha, China) with wide bandwidth and high sensitivity was selected to ensure reliable detection within the target frequency range. To preserve weak AE signals and improve the signal-to-noise ratio, an OPA627 operational amplifier (Texas Instruments, Dallas, TX, USA) was employed for signal conditioning, whose low noise and wide bandwidth are suitable for low-distortion amplification of transient signals. The conditioned signals were then digitized using an AD7356 analog-to-digital converter (Analog Devices, Wilmington, MA, USA), which provides 12-bit resolution, dual-channel simultaneous sampling, and a maximum sampling rate of 5 MSPS per channel, enabling accurate capture of transient AE waveform details. Finally, an SX1278 LoRa module (Semtech Corporation, Camarillo, CA, USA) was used for wireless transmission. Based on FSK modulation, the module offers strong anti-interference capability and reliable long-distance communication, making it suitable for distributed AE monitoring systems.

The AE nodes employ GTR150a sensors to passively detect high-frequency transient elastic waves induced by bolt corrosion. After analog front-end conditioning and digitization, the STM32F405RG microcontroller (STMicroelectronics, Geneva, Switzerland) performs signal buffering, event judgment, and data packet encapsulation. The processed data are then transmitted to the central gateway in a star topology via a 433 MHz LoRa wireless link based on the Semtech SX1278 chip. The host computer platform, developed using Python 3.8.10 and PyQt5, enables multi-node data display, storage, and export, thereby providing data support for subsequent feature extraction and corrosion classification.

**Figure 4:**
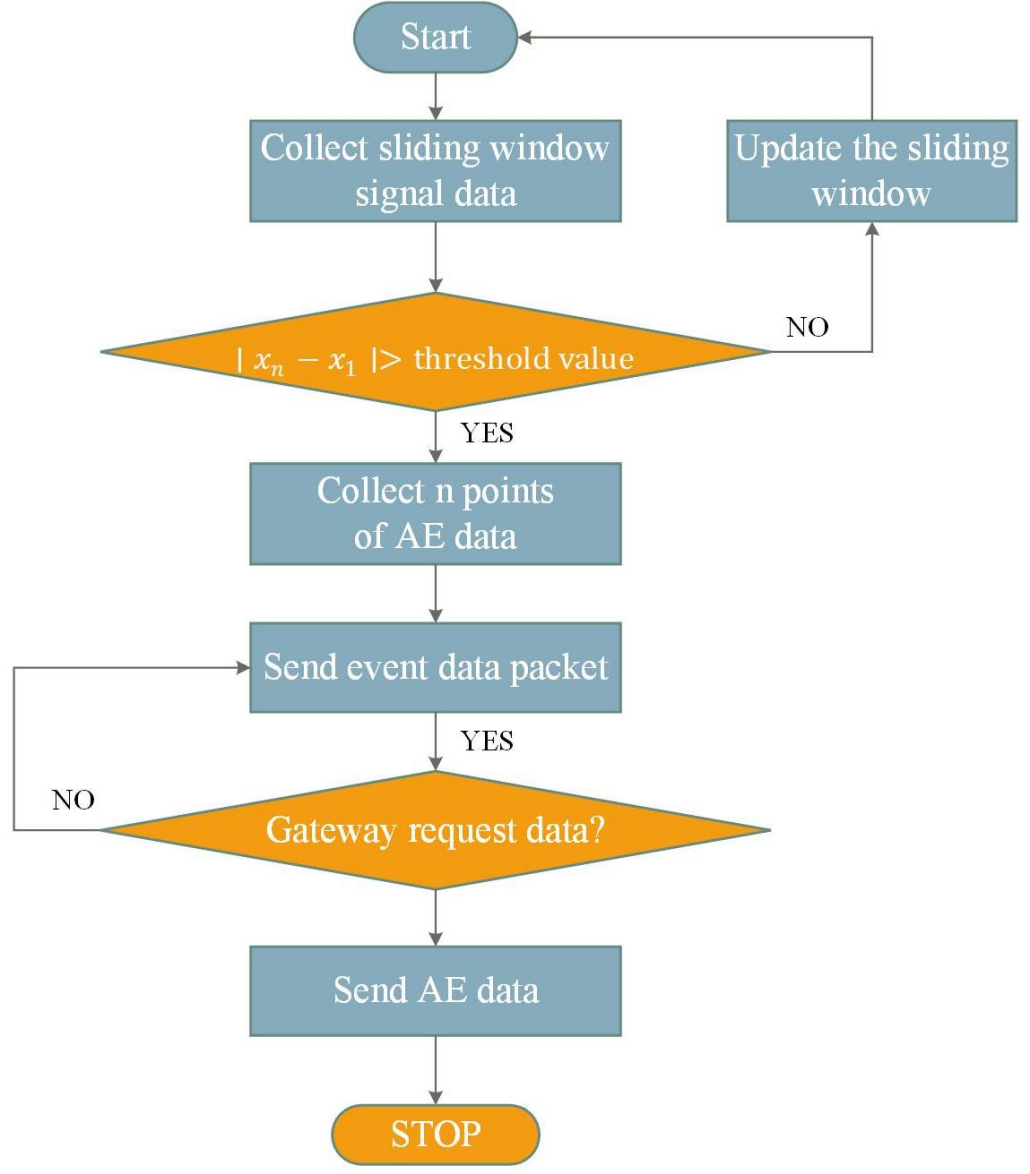
Main software flow of the AE acquisition node.

To achieve continuous online monitoring of AE activity, an event-triggered acquisition module is further integrated into the embedded software. The software execution flow is illustrated in Figure 4.

After system initialization, each node enters a cyclic monitoring state, in which AE signals are sampled in real time and evaluated using a sliding-window trigger strategy. Once an AE event is detected, the system captures the complete waveform segment and transmits the corresponding event packet. When the gateway issues a data request, the node returns either the buffered AE waveform packet or a no-event packet according to the cache status. After data transmission, the system returns to the signal sampling state, thereby realizing continuous online monitoring of AE signals.

Considering the burst-type and transient characteristics of AE signals, fixed-threshold detection is highly susceptible to background noise interference. Therefore, a sliding-window differential detection algorithm is adopted to improve both sensitivity and robustness. Specifically, a sliding window with a length of 30 is constructed to store the most recent sampled AE data. After each new sample is acquired, it is appended to the end of the window, while the earliest sample is removed. The signal mutation is then evaluated by calculating the difference between the first and last samples in the window:

(1) $$\Delta x=\left|x_{n}-x_{1}\right|$$

When the difference exceeds a predefined threshold, an AE event is considered to be triggered:

(2) $$\Delta x>T_{d}$$

where $x_{n}$ denotes the most recent sample in the window, $x_{1}$ denotes the earliest sample in the window, and $T_{d}$ is the differential threshold. In this study, the sliding-window length and differential threshold were set to 30 and 150, respectively. This approach can effectively detect abrupt AE signal variations while suppressing false triggering caused by background noise. In addition, to reduce transient interference during wireless communication, sampling was performed 500 ms after LoRa transmission.

### 2.2. Electrochemical Corrosion Experiments and Classification

In order to simulate the corrosion process of bolts in a typical marine or industrial environment and obtain AE signals under different corrosion degrees, this study uses the electrochemical accelerated corrosion method [38]. The high-strength bolt is immersed in NaCl solution as the anode and a constant electric field is applied to accelerate the electrochemical reaction. The main electrochemical reaction formula is as follows [39]:

(3) $$\mathrm{Fe}\to {\mathrm{Fe}}^{2+}+2e^{-}$$

(4) $$O_{2}+2H_{2}O+4e^{-}\to 4{\mathrm{OH}}^{-}$$

(5) $${\mathrm{Fe}}^{2+}+2{\mathrm{OH}}^{-}\to \mathrm{Fe}{\left(\mathrm{OH}\right)}_{2}$$

As the reaction proceeds, the resulting product further oxidizes into a loose, porous, reddish-brown rust. The volume expansion of the rust product triggers stress concentration within the material, leading to the initiation and propagation of microcracks, which in turn release transient elastic waves detectable by AE sensors.

To establish a mapping between corrosion severity and AE signals, this study referenced the international standards ISO 9227:2017 [40] and GB/T 16545-2015 [41] and divided the corrosion experiment into five stages (0 h, 6 h, 12 h, 18 h, and 24 h). Visual inspection and image analysis, together with ISO-based grading criteria, were used to assign these stages to five corrosion levels (0%, 25%, 50%, 75%, and 100%). As shown in Table 1, the five levels are associated with different surface appearance characteristics and corresponding ISO ratings, ranging from bright metallic sheen to severe rusting and pitting.

### 2.3. Multimodal Feature Fusion

**Figure 5:**
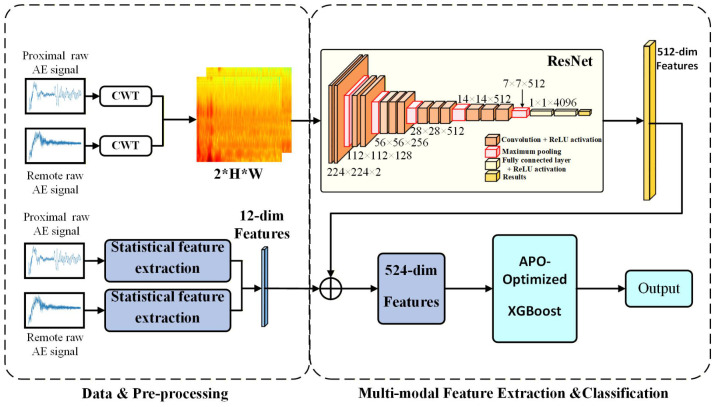
Multimodal fusion diagnosis model framework diagram.

To fully utilize the collaborative information from sensors at both ends of the bolt, this study proposes a multi-position, multi-modal feature fusion diagnostic framework. The overall process of this framework is shown in Figure 5. It mainly includes three parts: multimodal feature construction, feature fusion based on a dual-channel CNN, and a classifier based on APO-XGBoost.

**Figure 6:**
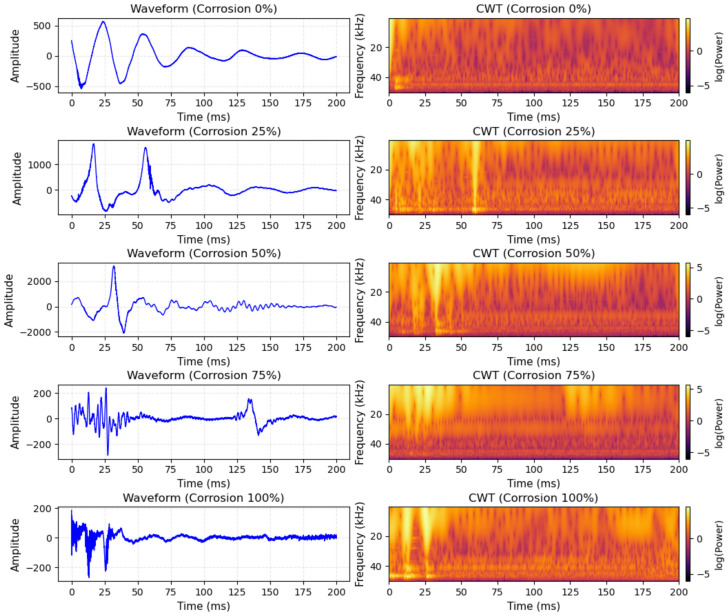
Typical AE signal waveforms and CWT time–frequency diagrams for different corrosion levels.

From the preprocessed raw AE signal, this study extracts two complementary characteristic modes:

**Time–frequency spectrum mode:** The continuous wavelet transform with the Morlet basis is applied to obtain a two-dimensional time–frequency representation of the one-dimensional AE signal [42]. To extract transient corrosion-related features from non-stationary acoustic emission signals, the continuous wavelet transform (CWT) with a Complex Morlet wavelet (cmor3-3) was employed for time–frequency analysis. The bandwidth parameter and center frequency were both set to 3, providing a suitable balance between time localization and frequency resolution. The scale range was set from 1 to 128 to characterize signal components from high-frequency transients to low-frequency attenuation. The resulting scalograms were resized to 224×224 pixels to match the input size of the convolutional neural network, and amplitude normalization was applied to reduce the influence of sensor gain variations. This graph can intuitively show the distribution of signal energy with time and frequency, effectively capturing the transient characteristics of AE signals [43]. Figure 6 shows the original AE waveform and its corresponding CWT time–frequency diagram under five different corrosion levels from 0% to 100%. As the corrosion level deepens, the energy distribution and high-frequency components in the CWT diagram show significant and regular changes, which provides a reliable data basis for subsequent deep learning feature extraction. The transformation formula is [44]

(6) $$W_{x}\left(a,b\right)=\frac{1}{\sqrt{a}}\int_{-\infty }^{+\infty }x\left(t\right)\psi^{*}\left(\frac{t-b}{a}\right)\;dt$$

where *a* is the scale factor, *b* is the translation factor, and ψ(t) is the Morlet mother wavelet.

**Statistical feature mode:** To preserve the signal’s global information and complement the time–frequency plot, this study extracts four key statistical metrics from the original one-dimensional signal: amplitude, duration, root mean square (RMS), and spectral kurtosis. Specifically, amplitude and duration are commonly used AE parameters for characterizing transient damage evolution [45]. RMS is adopted to describe the overall signal level, while kurtosis-related statistical information is used to characterize impulsive components in the signal [46]. Together, these features provide a compact description of transient behavior, signal level, and frequency-domain characteristics, and are further combined with the CWT-based time–frequency representation as multimodal inputs for subsequent classification. Such a design is intended to complement the discriminative information learned from raw temporal signals for fault diagnosis under noisy and varying operating conditions [47].

ResNet-18 was chosen as the backbone for deep feature extraction. ResNet introduces a residual block structure that uses shortcut connections to add input features element-by-element to the convolution output, thereby mitigating the effects of vanishing gradients and improving training stability. The output is equal to the element-wise addition of the convolution transformation result F(x) and the input *x*:

(7) H(x)=F(x)+x

This residual mapping transforms the learning objective from directly fitting H(x) to learning the more easily optimized F(x).

ResNet-18 was selected as the backbone network due to its strong feature extraction capability and relatively high computational efficiency, making it suitable for medium-scale CWT time–frequency image analysis [47]. To directly process the dual-channel information from the near- and far-end sensors, the first convolutional layer was modified from a 3-channel input to a 2-channel input. This allows the CWT time–frequency images from both sensors to be combined into a two-channel tensor, enabling the network to fuse multisource information at the input stage and to simultaneously examine signals from two spatial locations. This dual-channel time–frequency representation is then fed into the modified ResNet-18 network [48].

A pre-trained ResNet18 was adopted as the feature extractor, and only the last two residual blocks were fine-tuned to balance task adaptation and overfitting risk. The model was trained using the Adam optimizer with an initial learning rate of $1\times 10^{-4}$, a batch size of 32, and a weight decay of $1\times 10^{-4}$. In addition, a ReduceLROnPlateau scheduler based on the validation loss was employed, with a patience of 10 epochs and a decay factor of 0.5, while the remaining parameters were kept at their default settings.

After layer-by-layer feature extraction, the network produces a 512-dimensional deep feature vector containing rich spatial information [49]. In addition, 12 key statistical features were extracted from the near- and far-end signals, including their individual characteristics and differences. Finally, the 512-dimensional deep features were concatenated with the 12-dimensional statistical features to form a 524-dimensional fused feature vector.

### 2.4. APO-XGBoost-Based Classifier

To achieve accurate corrosion grade identification, this study employed Extreme Gradient Boosting (XGBoost) as the classifier and further introduced the Artificial Protozoa Optimizer (APO) to automatically optimize its key hyperparameters. As an efficient ensemble learning algorithm, XGBoost progressively improves prediction performance by iteratively constructing decision trees in a boosting manner. Its objective function combines the empirical loss and the model complexity regularization term, which helps suppress overfitting and enhance the generalization capability of the model [50]:

(8) $$L\left(\phi \right)=\sum_{i=1}^{n}l\left(y_{i},{\hat{y}}_{i}\right)+\sum_{k=1}^{K}\Omega \left(f_{k}\right)$$

where ϕ denotes the parameter set of all tree models, $l(y_{i},{\hat{y}}_{i})$ is the loss function measuring the discrepancy between the true label $y_{i}$ and the predicted value ${\hat{y}}_{i}$, and $\Omega (f_{k})$ represents the complexity penalty of the *k*th tree. During model construction, XGBoost adopts a greedy split strategy based on gain maximization, thereby generating an efficient and discriminative tree ensemble.

At the *t*th boosting iteration, the prediction can be written as

(9) $${\hat{y}}_{i}^{\left(t\right)}={\hat{y}}_{i}^{\left(t-1\right)}+f_{t}\left(x_{i}\right)$$

where ${\hat{y}}_{i}^{\left(t-1\right)}$ is the prediction of the previous (t−1) trees and $f_{t}\left(x_{i}\right)$ is the newly added tree. To efficiently optimize the objective, the loss function is approximated by a second-order Taylor expansion. After removing the constant term independent of $f_{t}$, the objective at the *t*th iteration can be expressed as

(10) $$L^{\left(t\right)}\approx \sum_{i=1}^{n}\left[g_{i}f_{t}\left(x_{i}\right)+\frac{1}{2}h_{i}f_{t}^{2}\left(x_{i}\right)\right]+\Omega \left(f_{t}\right)$$

where

(11) $$g_{i}=\frac{\partial \;l(y_{i},{\hat{y}}_{i}^{\left(t-1\right)})}{\partial {\hat{y}}_{i}^{\left(t-1\right)}},\;h_{i}=\frac{\partial^{2}\;l\left(y_{i},{\hat{y}}_{i}^{\left(t-1\right)}\right)}{\partial {\left({\hat{y}}_{i}^{\left(t-1\right)}\right)}^{2}}$$

denote the first- and second-order derivatives of the loss function, respectively. The regularization term of a tree is defined as

(12) $$\Omega \left(f_{t}\right)=\gamma T+\frac{1}{2}\lambda \sum_{j=1}^{T}w_{j}^{2}$$

where *T* is the number of leaf nodes, $w_{j}$ is the score of the *j*th leaf, γ is the penalty coefficient for the number of leaves, and λ is the L2 regularization coefficient. Accordingly, the optimal weight of each leaf node can be obtained as

(13) $$w_{j}^{*}=-\frac{\sum_{i\in I_{j}}g_{i}}{\sum_{i\in I_{j}}h_{i}+\lambda }$$

where $I_{j}$ denotes the set of samples assigned to the *j*th leaf node. These formulations allow XGBoost to efficiently evaluate split quality and improve classification performance while controlling model complexity.

**Figure 7:**
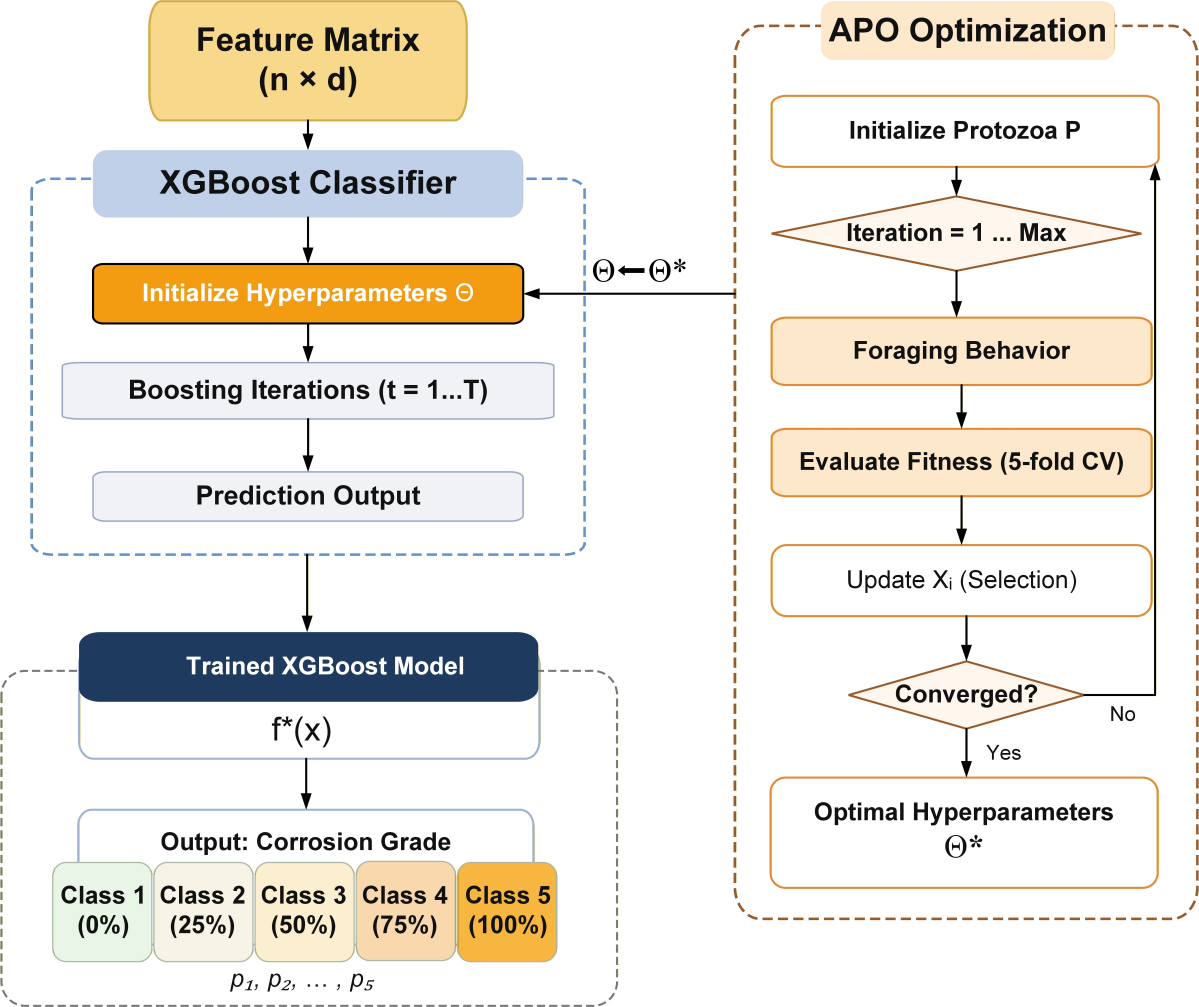
APO-XGBoost optimization framework. (* Indicates the optimal hyperparameters).

The performance of XGBoost is highly dependent on the selection of hyperparameters. Inappropriate parameter settings may lead to underfitting or overfitting and may significantly affect the classification accuracy and robustness. In the optimization process, the search space of each hyperparameter is predefined based on empirical knowledge and prior studies. In this study, XGBoost was configured for multi-class corrosion grade classification, and the softmax cross-entropy loss was adopted as the objective function. Therefore, to obtain a more reliable hyperparameter combination, this study introduced APO [51], a metaheuristic optimization algorithm derived from the biological behaviors of protozoa, including foraging, dormancy, and reproduction. By balancing global exploration and local exploitation, APO can efficiently search for promising solutions in a complex and nonlinear parameter space. The overall optimization procedure of the proposed APO-XGBoost framework is illustrated in Figure 7.

In the proposed APO-XGBoost framework, each protozoan individual in the APO population represents one candidate set of XGBoost hyperparameters. In the APO-based search, four XGBoost hyperparameters were tuned: the learning rate (learning_rate), maximum tree depth (max_depth), subsample ratio (subsample), and L2 regularization coefficient (reg_lambda). These hyperparameters are selected because they directly control the learning rate, model complexity, data sampling strategy, and regularization strength of XGBoost, thereby significantly influencing classification performance and generalization ability. In iteration *t*, the *i*th protozoan is represented by a position vector in the search space:

(14) $$X_{i}^{t}=\left[x_{i,1}^{t},x_{i,2}^{t},\ldots ,x_{i,d}^{t}\right]$$

where *d* is the number of hyperparameters to be optimized. Each dimension corresponds to one candidate hyperparameter of XGBoost. The fitness value corresponding to each protozoan is calculated by the average F1-score obtained from five-fold cross-validation:

(15) $$\mathrm{Fitness}\left(X_{i}^{t}\right)=\frac{1}{5}\sum_{m=1}^{5}F1_{m}$$

where $F1_{m}$ denotes the F1-score of the *m*th fold. The optimization objective is to maximize the fitness value, namely

(16) $$X^{*}=\arg\max_{X}\;\mathrm{Fitness}\left(X\right)$$

During the iterative search, APO updates the position of each protozoan according to the interaction between the current individual, the best-so-far solution, and stochastic exploration factors. A general update form can be expressed as

(17) $$X_{i}^{t+1}=X_{i}^{t}+\alpha r_{1}\left(X_{best}^{t}-X_{i}^{t}\right)+\beta r_{2}\left(X_{rand}^{t}-X_{i}^{t}\right)$$

where $X_{best}^{t}$ is the best individual in the current population, $X_{rand}^{t}$ is a randomly selected individual, $r_{1}$ and $r_{2}$ are random numbers in [0,1], and α and β are adaptive control parameters. Through iterative position updating and fitness evaluation, APO gradually converges to the hyperparameter combination that yields the best classification performance. Therefore, the APO-XGBoost model can effectively improve the robustness and recognition accuracy of corrosion grade classification.

The APO algorithm is used to optimize four key hyperparameters of XGBoost, including learning_rate, max_depth, subsample, and reg_lambda. The search ranges and the corresponding optimal parameter combination obtained by APO are summarized in Table 2.

## 3. Results

**Figure 8:**
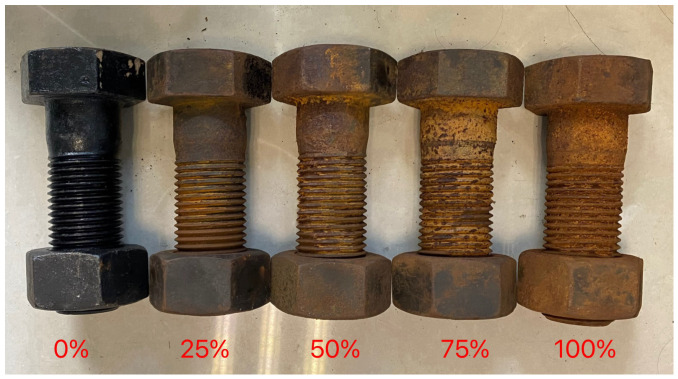
Bolt morphology of five corrosion levels.

We used five M52, grade 10.9 high-strength bolts as experimental subjects. Electrochemically accelerated corrosion was performed in a 3.5% NaCl solution to simulate five corrosion levels (0%, 25%, 50%, 75%, and 100%). The physical morphologies are shown in Figure 8. After the corrosion steps, the bolt surfaces were cleaned and dried to remove residual solution and prevent interference with AE signal acquisition.

**Figure 9:**
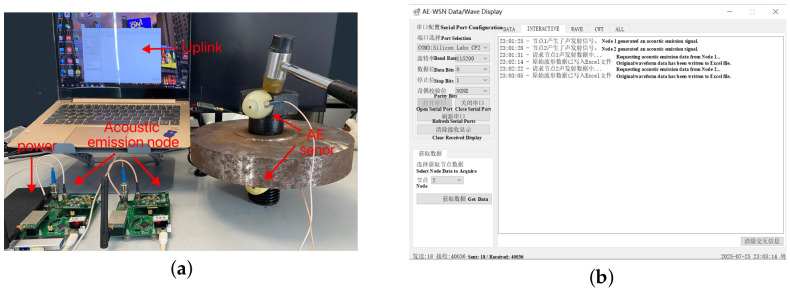
(**a**) Experimental scene layout; (**b**) wireless data transmission host computer.

To collect raw acoustic emission signals, two GTR150a sensors were mounted on the screw head and nut sides, respectively, as shown in Figure 9. A rubber hammer was used as the excitation source. The signals were collected via a wireless node module and uploaded to a host computer. The two AE sensors used a coupling medium to enhance signal transmission quality, and the sensors were placed vertically on either side of the flange plate to ensure consistent signal acquisition. Finally, 100 AE signal records were collected on each sensor device for each corrosion level, forming a record containing 1000 samples. Each sample was collected at a frequency of 50 kHz and had a length of 10,000 points.

### 3.1. Overall Performance of the Multimodal Fusion Model

Since the two AE sensors are fixed to the bolt head (near-end) and the nut (far-end), respectively, there are significant differences in their distances from the potential corrosion core area and in their signal propagation paths. This positional difference causes the AE signals collected from the two sensors to exhibit noticeable differences in amplitude, attenuation characteristics, and frequency response. To ensure the fairness of the evaluation, the dataset was divided into training and test sets in a 7:3 ratio using stratified random sampling. Accuracy, precision, recall, and F1-score were used as the core evaluation metrics [52]. During the APO-based hyperparameter optimization process, five-fold cross-validation was employed to evaluate the fitness of candidate solutions.

(18) $$\mathrm{Accuracy}=\frac{TP+TN}{TP+TN+FP+FN}$$

(19) $$\mathrm{Precision}=\frac{TP}{TP+FP}$$

(20) $$\mathrm{Recall}=\frac{TP}{TP+FN}$$

(21) $$F1=\frac{2\times \mathrm{Precision}\times \mathrm{Recall}}{\mathrm{Precision}+\mathrm{Recall}}$$

The intelligent diagnosis model based on multimodal feature fusion and APO-XGBoost (MMF-XGBoost) uses ResNet-18 to extract deep features from cascaded dual-channel CWT time–frequency maps, fuses key statistical features, and ultimately performs classification using XGBoost optimized by the APO algorithm. This model was evaluated on the test set, and the performance metrics are shown in Table 3.

**Figure 10:**
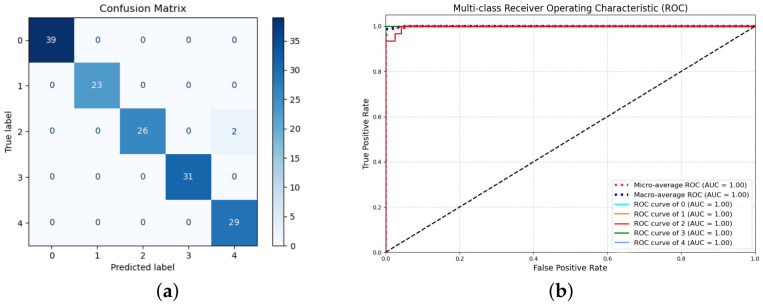
MMF-XGBoost model: (**a**) confusion matrix; (**b**) ROC curve.

In Table 3, the MMF-XGBoost model achieved a high accuracy of 98.67% and an F1-score of 0.9855. This demonstrates that the model effectively integrates information from different sensors, providing highly robust recognition results even with varying sensor locations and signal fluctuations. The model performance was further analyzed using the confusion matrix and receiver operating characteristic (ROC) curves, as illustrated in Figure 10. The ROC curves for all corrosion levels are concentrated near the upper-left corner, and the AUC values are close to 1.00, indicating strong discriminative capability of the proposed model.

### 3.2. Ablation Studies and Component Analysis

To further verify the effectiveness of the proposed framework, a series of comparative and ablation experiments were conducted. For fair comparison, all CNN backbone networks were trained under identical experimental settings, including the same number of training epochs, learning rate, optimizer, data augmentation strategy, input preprocessing pipeline, and evaluation protocol. In addition, the effects of classifier choice and hyperparameter optimization were systematically investigated under consistent settings. The results on the test set are summarized in Table 4.

As shown in Table 4, ResNet18 + APO-XGBoost achieved the best overall performance among all compared methods. It consistently outperformed the baseline models, demonstrating the effectiveness of the ResNet18 backbone and the APO-based optimization strategy for multimodal AE signal recognition.

**Figure 11:**
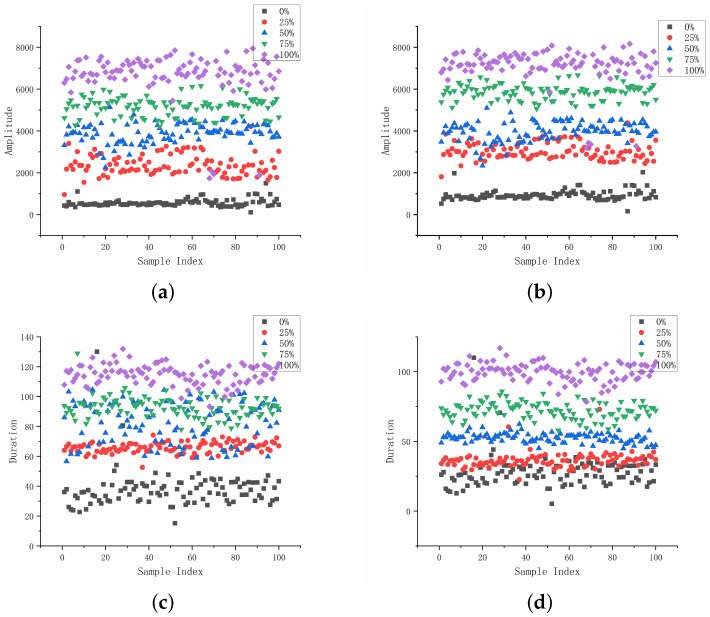
Scatter distribution of amplitude and duration at different corrosion levels: (**a**) near-end amplitude, (**b**) far-end amplitude characteristics, (**c**) near-end duration, and (**d**) far-end duration.

The scatter distributions of amplitude and duration at different corrosion levels are shown in Figure 11. With increasing corrosion level, the distributions of both features gradually shift toward higher values for the two sensors. Low-corrosion samples are mainly located in lower-value regions, whereas higher corrosion levels tend to appear in higher ranges. Despite partial overlap between adjacent levels, a relatively clear separation can still be observed, indicating that amplitude and duration are informative for corrosion-stage discrimination.

To evaluate the effect of time–frequency representation on classification performance, additional experiments were conducted using WT-based and CWT-based dual-channel inputs. Specifically, the near-end and far-end acoustic emission signals were converted into time–frequency images, stacked as two input channels, and fed into the same ResNet-18 backbone for classification.

As shown in Table 5, the CWT-based dual-channel input achieved better performance than the WT-based counterpart across all evaluation metrics. Therefore, the CWT-based representation was adopted in the subsequent fusion strategy analysis.

**Figure 12:**
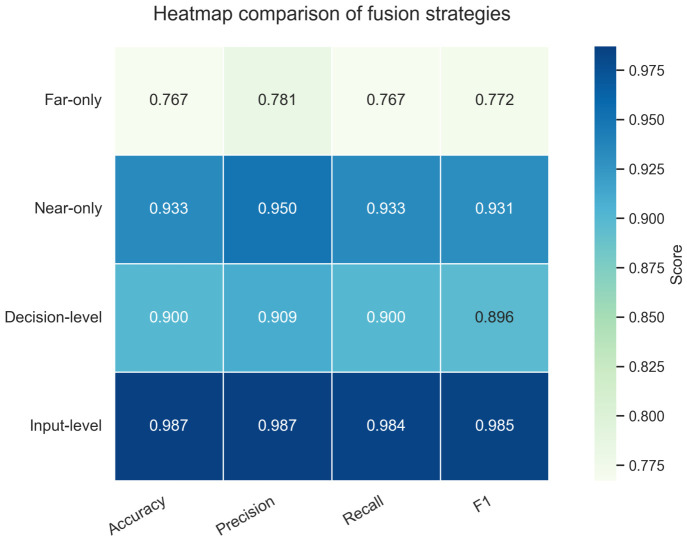
Heatmap comparison of different fusion strategies in terms of accuracy, precision, recall, and F1-score.

To examine whether the performance improvement is related to the use of multisource information or to the fusion strategy, additional experiments were conducted using the near-end and far-end CWT time–frequency images. Far-only, near-only, decision-level fusion, and the proposed input-level fusion were compared. As shown in Figure 12, the near-only setting outperformed the far-only setting, indicating that the near-end sensor provides more discriminative corrosion information.

In the decision-level fusion setting, the prediction probabilities of two separately trained models were combined at the output stage. Although this strategy improved performance relative to the far-only setting, it still underperformed the near-only setting. By contrast, the input-level fusion strategy achieved the best results across all evaluation metrics, indicating that it most effectively exploits the complementary information from the two sensor signals.

To further assess the robustness of the proposed method under noisy conditions, Gaussian white noise at different SNR levels was superimposed on the raw AE signals before CWT transformation.

**Figure 13:**
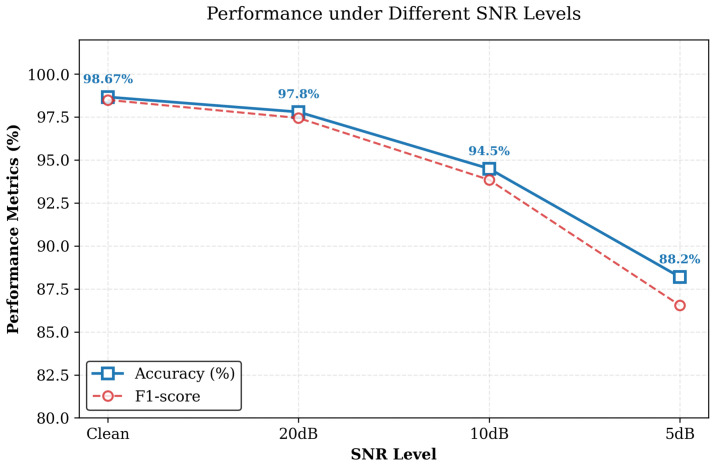
Performance comparison under different SNR levels.

As shown in Figure 13, the proposed method maintains relatively stable performance under the 20 dB and 10 dB conditions. In particular, the classification accuracy remains 94.50% at 10 dB, indicating good robustness to moderate noise interference. When the SNR decreases to 5 dB, the classification accuracy drops to 88.20%, indicating that severe noise has a more pronounced impact on feature representation and subsequent classification performance.

Overall, both accuracy and F1-score decrease with decreasing SNR, with a more pronounced degradation observed at 5 dB.

### 3.3. Model Interpretability Analysis

**Figure 14:**
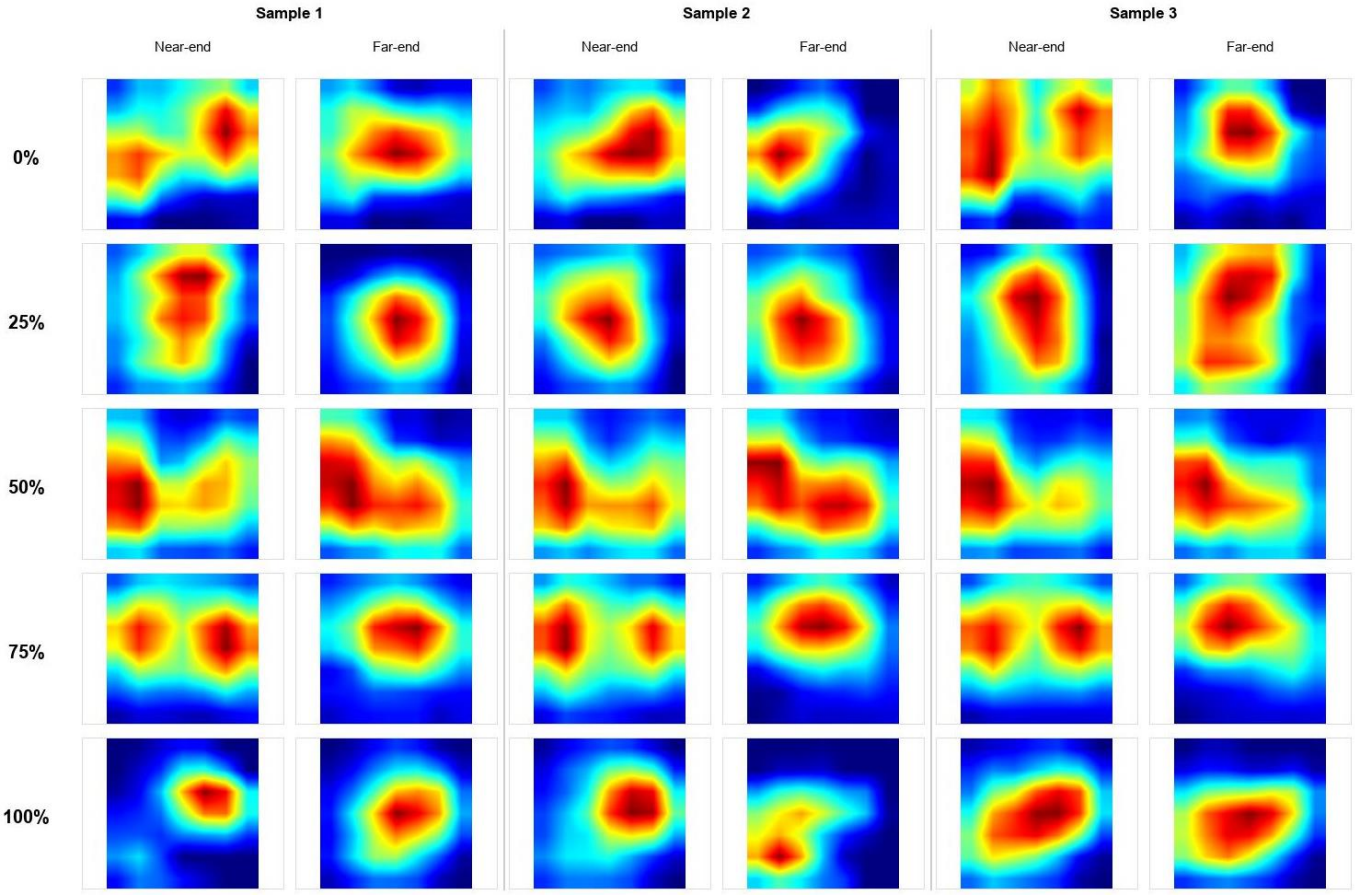
Grad-CAM heatmap comparison across corrosion levels. (Red/yellow indicates higher attention, while green/blue indicates lower attention).

To further investigate why the proposed MMF-XGBoost model can effectively perform corrosion diagnosis, an interpretability analysis was conducted from two perspectives: feature extraction and classification decision-making. Grad-CAM was employed to visualize the decision basis of the modified ResNet-18. Since the model takes dual-channel inputs (one channel corresponding to the near-end CWT and the other to the far-end CWT), Grad-CAM produces a unified attention heatmap of size 224 × 224. To intuitively identify which sensor contributes most to the discriminative features, the same heatmap was overlaid onto the CWT background images of both the near-end and far-end signals. Figure 14 shows the Grad-CAM visualization results.

The results show that in the healthy state (0%), the model’s highlighted activation regions are primarily concentrated in the high-frequency region on the left side of the time–frequency plot. As corrosion progresses (25%–50%), the model’s attention shifts to the mid- and low-frequency bands, and the newly highlighted regions are more concentrated in the far-end CWT plot. In the severe corrosion stage (75%), attention simultaneously covers the high-frequency initial impulse at the near-end and the mid-frequency response at the far-end. At 100% complete corrosion, the model’s attention refocuses on the late-stage response region of the near-end signal.

To further support the Grad-CAM observations, the evolution of acoustic emission signals with increasing corrosion level was quantitatively analyzed in terms of frequency-band energy and frequency centroid. Based on the CWT-derived time–frequency representation, the frequency domain was divided into eight sub-bands (B1–B8) from low to high frequency. The normalized energy ratio in each band was calculated from the corresponding CWT coefficients, where B1–B2, B3–B5, and B6–B8 denote the low-, mid-, and high-frequency ranges, respectively. All values were averaged over the samples at each corrosion level.

**Figure 15:**
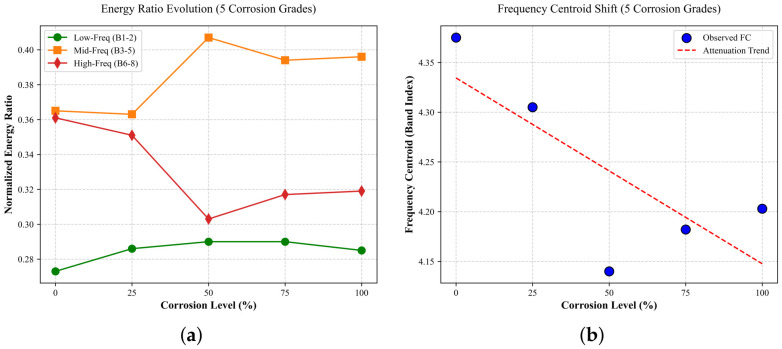
Evolution of frequency-band energy ratios and frequency centroid under different corrosion levels: (**a**) normalized energy ratio in different frequency bands; (**b**) frequency centroid variation.

As shown in Figure 15, the mid-frequency band accounts for the largest energy proportion around the 50% corrosion level, whereas the high-frequency energy shows an overall decreasing trend with increasing corrosion level and the low-frequency energy remains relatively stable. Meanwhile, the frequency centroid shows an overall downward trend, suggesting a gradual reduction in the contribution of high-frequency components.

**Figure 16:**
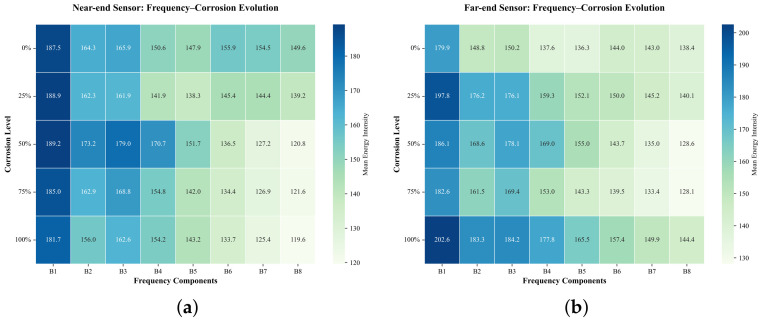
Frequency-band energy distributions under different corrosion levels for the two sensors: (**a**) near-end sensor; (**b**) far-end sensor.

As shown in Figure 16, the energy distribution across frequency bands varies with corrosion level for both sensors. Noticeable differences between the near-end and far-end sensors are also observed, particularly in the mid- to high-frequency bands, indicating that sensor position affects AE signal energy distribution.

Grad-CAM provides a qualitative interpretation of the model attention. The observed reduction in high-frequency energy and the downward shift of the frequency centroid are consistent with the attenuation characteristics of corrosion-induced AE signals. Meanwhile, the differences between the two sensors further support the effectiveness of the proposed dual-channel fusion strategy in capturing complementary information.

**Figure 17:**
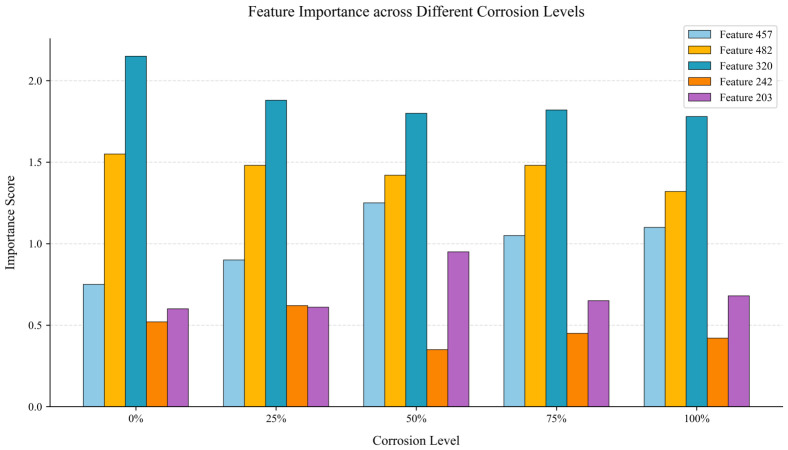
Comparison of the top five important features across different corrosion levels.

To understand how the APO-XGBoost classifier utilizes the extracted 524-dimensional fused features to make decisions, this study analyzed feature importance in XGBoost. Figure 17 shows the five features that contribute most to each corrosion grade assessment, along with their importance scores.

### 3.4. APO Algorithm Performance Verification

To demonstrate the advantages of the APO algorithm used in this study for hyperparameter optimization, this section evaluates its performance through independent benchmarks. We selected four standard test functions commonly used in optimization, including the simple unimodal Sphere function and the complex multimodal Rastrigin, Griewank, and Schwefel functions. APO was also compared with several mainstream optimization algorithms, such as particle swarm optimization (PSO), genetic algorithm (GA), and random search (RS).

**Figure 18:**
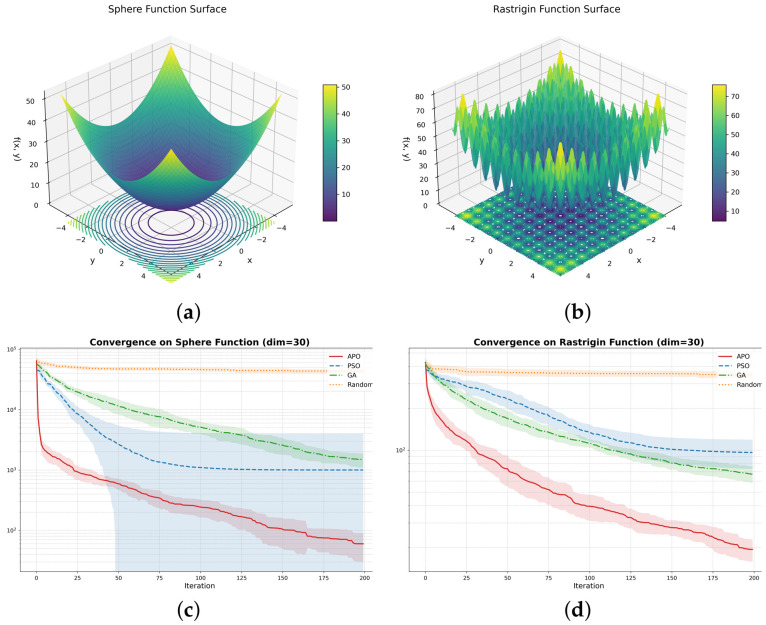

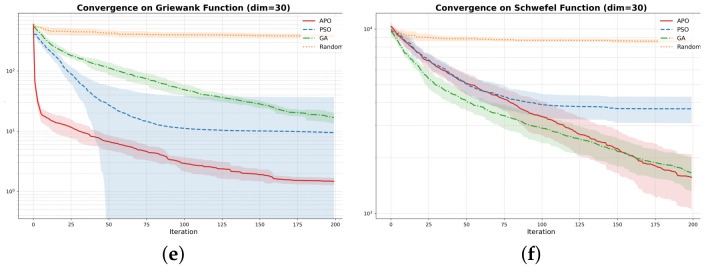
Visualization of benchmark functions and comparison of the convergence performance of various optimization algorithms: (**a**) single-peak Sphere function surface; (**b**) complex multi-peak Rastrigin function surface; (**c**–**f**) convergence curves of each algorithm on the Sphere, Rastrigin, Griewank, and Schwefel functions, respectively.

As shown in Figure 18, the benchmark results indicate that APO achieves competitive overall optimization performance. On the Sphere, Rastrigin, and Griewank functions, APO converges to lower objective values than the other compared algorithms. On the Schwefel function, both APO and GA perform well and clearly outperform PSO and random search, demonstrating the strong global search capability of APO.

In addition to standard benchmark functions, APO was further compared with other optimization algorithms to evaluate its hyperparameter optimization capability in a practical engineering scenario. Under the same experimental conditions and using the same fused feature input, APO, Bayesian Optimization (BO), Particle Swarm Optimization (PSO), and Random Search (RS) were applied to the hyperparameter search of XGBoost. BO, PSO, and RS were selected as representative comparison methods, corresponding to surrogate-based optimization, classical population-based meta-heuristic search, and unguided random exploration, respectively. Their mean performance under five-fold cross-validation was then compared in terms of classification accuracy, F1-score, convergence behavior, and average search time.

**Figure 19:**
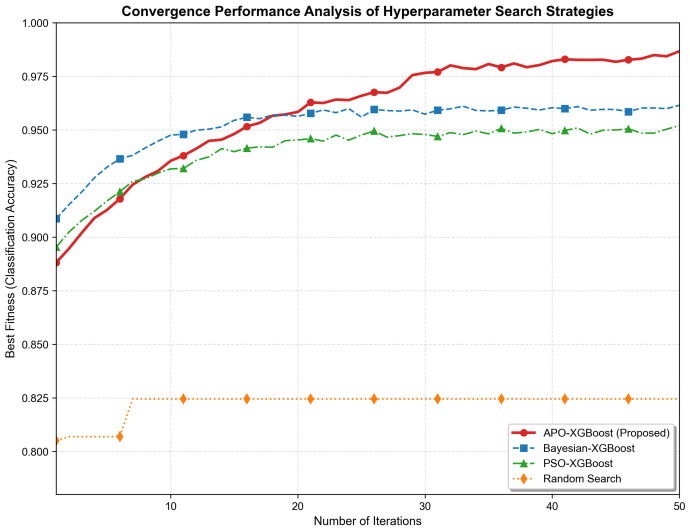
Optimization process comparison of different hyperparameter optimization strategies on the bolt corrosion classification task.

Figure 19 illustrates the convergence processes of different hyperparameter optimization methods on the bolt corrosion classification task. BO exhibits relatively rapid performance improvement during the early iterations, whereas APO maintains sustained optimization in the middle and later stages and ultimately achieves the highest classification accuracy. PSO converges more slowly and its final performance remains lower than that of APO and BO. In contrast, Random Search adopts a non-iterative random sampling mechanism, and therefore its performance exhibits a step-like pattern rather than a continuous convergence trend.

To more meaningfully assess the robustness of different optimization strategies, quantitative statistical evaluation was further conducted on the practical bolt corrosion classification task. Specifically, five-fold cross-validation was performed on the classification model under the same experimental settings. Each optimization strategy was applied to search the hyperparameters within the same cross-validation framework, and the mean accuracy, F1-score, average search time, as well as the corresponding best accuracy and standard deviation are summarized in Table 6.

Table 6 and Figure 19 show that different hyperparameter optimization methods exhibit clear performance differences on the bolt corrosion classification task. APO achieved the best overall classification performance in terms of mean accuracy, F1-score, best accuracy, and standard deviation among the compared methods, although BO required less average search time. PSO converged more slowly in this experiment, with limited performance improvement in the later stage, and its final performance remained lower than that of APO and BO. Random Search, which mainly relies on random sampling and lacks effective utilization of historical search information, resulted in substantially lower overall performance. Combined with the convergence trends and quantitative results, these findings indicate that APO is an effective and stable hyperparameter optimization strategy for XGBoost in this task.

### 3.5. Cross-Specification Generalization Validation

**Figure 20:**
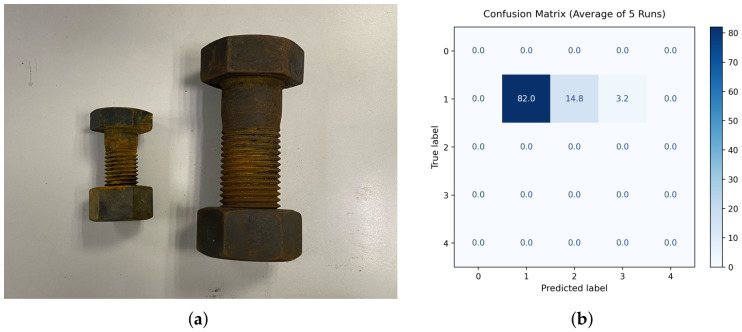
(**a**) Example of an M30 bolt specimen with 25% corrosion. (**b**) Averaged confusion matrix of the cross-specification validation results on the unseen M30 dataset over five independent runs.

To evaluate the generalization and robustness of the proposed model across different bolt specifications, a cross-specification validation experiment was conducted using M30 high-strength bolts. Based on the best-performing model obtained in the previous experiments under the 5-fold cross-validation framework on the M52 dataset, cross-specification testing was further carried out on the unseen M30 dataset without any additional fine-tuning or parameter updates. In this experiment, 100 AE signal samples with a 25% corrosion level were collected from M30 bolts for testing. To reduce the influence of random variation, the evaluation on the unseen M30 dataset was repeated five times, and the averaged results are reported. An example of the M30 bolt specimen is shown in Figure 20a.

The classification results for the M30 dataset are shown in Figure 20b. The fixed model achieved an average accuracy of 82.0% on the unseen M30 dataset, with a standard deviation of 1.3% over five independent runs, indicating relatively stable cross-specification performance. As shown in the averaged confusion matrix, 82.0% of the 25% corrosion samples were correctly classified into the corresponding category, while 14.8% were misclassified as 50% and 3.2% as 75%. No samples were misclassified as 0% or 100%. These results suggest that the proposed model retains reasonable discrimination ability when transferred from the M52 specification to the unseen M30 specification, although the results also indicate a tendency for the smaller-sized bolts to be predicted as having relatively higher corrosion severity.

It should be noted that this validation is still preliminary, since only M30 samples with a 25% corrosion level were evaluated. Future work will further examine a wider range of bolt specifications and corrosion levels to more comprehensively assess the general applicability of the proposed framework.

## 4. Conclusions

This study proposed a bolt corrosion diagnosis framework (MMF_APO_XGBoost) based on multimodal feature fusion and optimized machine learning. Dual-channel CWT representations of near-end and far-end AE signals were used as inputs, allowing complementary information from different sensor positions to be integrated at the early input stage. The model achieved an accuracy of 98.67% in the experiment, indicating strong classification capability for bolt corrosion states. Grad-CAM results further showed that the discriminative regions shifted from high-frequency components to lower-frequency responses as corrosion progressed, which is consistent with the attenuation characteristics of corrosion-induced AE signals. A preliminary cross-specification test on unseen M30 bolts also demonstrated that the model retained reasonable prediction capability beyond the training specification.

Future research will extend the dataset to bolts with different specifications and materials, conduct leave-one-specification-out validation experiments, and investigate model robustness under lower signal-to-noise conditions. These efforts aim to further improve cross-specification generalization and practical applicability of the proposed framework.

## Figures and Tables

**Table 1:** Corrosion grade–morphology–ISO rating comparison table.

Severity	Visual Appearance	ISO Rating
0%	Bright metallic sheen	10
25%	Light oxidation, few small rust spots	7
50%	Moderate corrosion; discoloration and extensive formation	5
75%	Severe rust, localized flaking	3
100%	Extreme rusting, pitting, including cracking or delamination	1

**Table 2:** Search ranges and optimal hyperparameters of XGBoost optimized by APO.

Hyperparameter	Search Range	Optimal Value
learning_rate	[0.01, 0.30]	0.1110
max_depth	[3, 10]	7
subsample	[0.5, 1.0]	0.7816
reg_lambda	[0, 5]	1

**Table 3:** Overall performance of the MMF-XGBoost model.

Method	Accuracy (%)	Precision (%)	Recall (%)	F1-Score
MMF-XGBoost	98.67	98.71	98.40	0.9855

**Table 4:** Performance comparison of different backbones, classifiers, and optimization strategies on the test set. (Bold values indicate the best results).

Category	Model/Framework	Accuracy (%)	F1-Score	Test Loss
Backbone	AlexNet + APO-XGB	94.13	0.9385	0.0053
	MobileNetV2 + APO-XGB	95.50	0.9520	0.0026
	VGG19 + APO-XGB	95.91	0.9575	0.0031
	GoogLeNet + APO-XGB	96.47	0.9610	0.0035
	**ResNet18 + APO-XGB**	**98.67**	**0.9855**	**0.0021**
Classifier	ResNet18 (CNN)	95.33	0.9510	0.0654
	ResNet18 + SVM	82.33	0.8291	N/A
	ResNet18 + RF	89.45	0.8977	N/A
Optimization	ResNet18 + XGB (Def.)	96.85	0.9650	0.0152
	**ResNet18 + APO-XGB**	**98.67**	**0.9855**	**0.0021**

**Table 5:** Performance comparison of WT-based and CWT-based dual-channel time–frequency representations for bolt corrosion classification.

Method	Accuracy (%)	Precision (%)	Recall (%)	F1-Score
WT dual-channel	94.20	94.35	94.10	0.9422
CWT dual-channel	98.67	98.71	98.40	0.9855

**Table 6:** Performance and statistical comparison of different hyperparameter optimization strategies for XGBoost under five-fold cross-validation on the bolt corrosion classification task.

Search Strategy + XGBoost	Mean Accuracy (%)	F1-Score (%)	Avg. Search Time (s)	Best Accuracy (%)	Std. Dev. (%)
APO	98.67	98.76	452.3	99.12	0.24
BO	96.15	96.28	280.5	96.80	0.45
PSO	95.20	95.37	592.8	95.90	0.62
RS	82.45	82.77	315.2	85.10	2.10

## Data Availability

Data are available from the corresponding author upon reasonable request.

## References

[1] Wang C., Zhang W.-W., Wang H.-R. (2020). Experimental Study on Crack Detection at Bolt Hole Edge in Rail Joint Using the Third Harmonic. J. Phys. Conf. Ser..

[2] Wen J., Li Z., Hu T., Liu L. (2018). Simple analysis on failure of high strength bolts in Chongqing Chaotianmen Bridge. IOP Conf. Ser. Mater. Sci. Eng..

[3] Daniel E.F., Wang C., Li C., Dong J., Udoh I.I., Zhang D., Zhong W., Zhong S. (2023). Evolution of corrosion degradation in galvanised steel bolts exposed to a tropical marine environment. J. Mater. Res. Technol..

[4] Zhu N., Jin F., Kong X., Xu Y., Zhou J., Wang B., Wu H. (2018). Interface and anti-corrosion properties of sea-sand concrete with fumed silica. Constr. Build. Mater..

[5] Biezma M., Andrés M., Agudo D., Briz E. (2020). Most fatal oil & gas pipeline accidents through history: A lessons learned approach. Eng. Fail. Anal..

[6] Wu S., Ma X., Zhang X., Chen J., Yao Y., Li D. (2024). Investigation into hydrogen induced fracture of cable bolts under deep stress corrosion coupling conditions. Tunn. Undergr. Space Technol..

[7] He Z., Zhang N., Xie Z., Ma C., Han C., Zhang F., Wang H., Alarifi S.S. (2025). Study on stress corrosion behavior and failure mechanism of galvanized bolts in complex coal mine environments. J. Mater. Res. Technol..

[8] Villalba E., Atrens A. (2008). Metallurgical aspects of rock bolt stress corrosion cracking. Mater. Sci. Eng. A.

[9] Wu S., Hao W., Yao Y., Li D. (2023). Investigation into durability degradation and fracture of cable bolts through laboratorial tests and hydrogeochemical modelling in underground conditions. Tunn. Undergr. Space Technol..

[10] Guo Y., Hu Z., Xiong L., Zhou X., Zhu P. (2022). Fiber Bragg grating based quasi-distributed bolt force sensor with torque resistance. Measurement.

[11] Herbst F., Chadda R., Peters J., Riehl D., Hartmann C., Suppelt S., Breimann R., Kirchner E., Hofmann K., Matthiesen S. (2024). Sensor-integrating Bolt for Multi-axial Force Measurement. IEEE Sens. J..

[12] Xu L., Shi S., Huang Y., Yan F., Wang X., Wilson R., Zhang D. (2025). Quantification and assessment of steel pitted corrosion using optical frequency domain reflectometry (OFDR)-based distributed fiber optic sensors. Meas. J. Int. Meas. Confed..

[13] Ahn J.-H., Lee J.M., Cheung J.-H., Kim I.-T. (2016). Clamping force loss of high-strength bolts as a result of bolt head corrosion damage: Experimental research A. Eng. Fail. Anal..

[14] Shah J., Wang H., Mukherjee A. (2025). Investigating the Correlation Between Corrosion-Induced Bolt Head Damage and Preload Loss Using Ultrasonic Testing. Sensors.

[15] Zhang N., Huang J., Zhang X., Chen N., Jiang Y. (2022). Intelligent Cloud Bolt for High-Precision Pretightening force Monitoring and Preparation Method. Chinese Patent Application.

[16] Sheikh M.F., Kamal K., Rafique F., Sabir S., Zaheer H., Khan K. (2021). Corrosion detection and severity level prediction using acoustic emission and machine learning based approach. Ain Shams Eng. J..

[17] Pan T., Zheng Y., Zhou Y., Luo W., Xu X., Hou C., Zhou Y. (2023). Damage pattern recognition for corroded beams strengthened by CFRP anchorage system based on acoustic emission techniques. Constr. Build. Mater..

[18] Han G., Lv S., Tao Z., Sun X., Du B. (2024). Evaluation of Bolt Corrosion Degree Based on Non-Destructive Testing and Neural Network. Appl. Sci..

[19] Li L., Yuan B., Liu L., Song R. (2025). Intelligent Damage Detection for High Strength Bolts on Wind Turbines Based on Deep Cognitive Method. IEEE Access.

[20] Kralovec C. (2023). Artificial Intelligence-Based Corrosion Sensing and Prediction for Aircraft Applications (AICorrSens).

[21] Zhou Y., Liang M., Yue X. (2024). Deep residual learning for acoustic emission source localization in a steel–concrete composite slab. Constr. Build. Mater..

[22] Ju S., Li M., Fang C.Z., Wang M.H. (2020). Identification of wood damage processes based on acoustic emission signals at instantaneous frequencies. J. N. For. Univ..

[23] Grosse C.U., Ohtsu M., Aggelis D.G., Shiotani T. (2021). Acoustic Emission Testing: Basics for Research–Applications in Engineering.

[24] Ohtsu M. (2020). Acoustic Emission and Related Non-Destructive Evaluation Techniques in the Fracture Mechanics of Concrete: Fundamentals and Applications.

[25] Krautkrämer J., Krautkrämer H. (2013). Ultrasonic Testing of Materials.

[26] Dai L., Wang L. (2007). Magnetic Particle Inspection of High-Temperature Fastening Bolts in Thermal Power Plants. Nondestruct. Test..

[27] Lovejoy M. (1993). Magnetic Particle Inspection: A Practical Guide.

[28] Yao K., Wang Z., Deng B., Shen K. (2012). Experimental research on metal magnetic memory method. Exp. Mech..

[29] Park S., Yun C.-B., Roh Y., Lee J.-J. (2006). PZT-based active damage detection techniques for steel bridge components. Smart Mater. Struct..

[30] Tian S. (2021). Fracture Monitoring of High-Strength Bolts Based on Acoustic Emission Technology. Master’s Thesis.

[31] Gao X., Wang W., Du J. (2024). Bolt load looseness detection for slip-critical blind bolt based on wavelet analysis and deep learning. Structures.

[32] Li S., He R., Wu Y., Yang Y., Wu G. (2026). Machine learning-assisted acoustic emission localization of simulated wire-break events in prestressed strands of high-speed railway box girders. Adv. Eng. Inform..

[33] Madhusudana C.K., Kumar H., Narendranath S. (2018). Fault Diagnosis of Face Milling Tool using Decision Tree and Sound Signal. Mater. Today Proc..

[34] Zhang Z., Xiao Y., Su Z., Pan Y. (2019). Continuous monitoring of tightening condition of single-lap bolted composite joints using intrinsic mode functions of acoustic emission signals: A proof-of-concept study. Struct. Health Monit..

[35] Wang R., Zhang P., Zhang L., Wang H., Zhang L. (2024). Corrosion State Monitoring Based on Multi-Granularity Synergistic Learning of Acoustic Emission and Electrochemical Noise Signals. Processes.

[36] Haitz D., Jutzi B., Huebner P., Ulrich M. (2022). Corrosion detection for industrial objects: From multi-sensor system to 5D feature space. arXiv.

[37] Chernov A.V., Butusov A.N., Vasiliev M.A., Zimenkov A.A., Pesterev D.O. (2022). Integrated Video and Acoustic Emission Data Fusion for Intelligent Decision Making in Material Surface Inspection System. Sensors.

[38] Yu A.-P., Naqvi M.W., Hu L.-B., Zhao Y.-L. (2020). An experimental study of corrosion damage distribution of steel bars in reinforced concrete using acoustic emission technique. Constr. Build. Mater..

[39] Jones D.A. (1996). Principles and Prevention of Corrosion.

[40] (2017). Corrosion Tests in Artificial Atmospheres—Salt Spray Tests.

[41] (2015). Corrosion of Metals and Alloys—Removal of Corrosion Products from Corrosion Test Specimens. General Administration of Quality Supervision, Inspection and Quarantine of the People’s Republic of China.

[42] Lu C., Ding P., Chen Z. (2011). Time-frequency analysis of acoustic emission signals generated by tension damage in CFRP. Procedia Eng..

[43] Hong J.C., Sun K.H., Kim Y.Y. (2005). Dispersion-based short-time Fourier transform applied to dispersive wave analysis. J. Acoust. Soc. Am..

[44] Mallat S. (2008). A Wavelet Tour of Signal Processing: The Sparse Way.

[45] Ebrahimkhanlou A., Choi J., Hrynyk T.D., Salamone S., Bayrak O. (2020). Acoustic emission monitoring of containment structures during post-tensioning. Eng. Struct..

[46] Zhan L., Ma F., Zhang J., Li C., Li Z., Wang T. (2019). Fault feature extraction and diagnosis of rolling bearings based on enhanced complementary empirical mode decomposition with adaptive noise and statistical time-domain features. Sensors.

[47] Zhang W., Li C., Peng G., Chen Y., Zhang Z. (2018). A deep convolutional neural network with new training methods for bearing fault diagnosis under noisy environment and different working load. Mech. Syst. Signal Process..

[48] Wang J., Wang D., Wang S., Li W., Song K. (2021). Fault Diagnosis of Bearings Based on Multi-Sensor Information Fusion and 2D Convolutional Neural Network. IEEE Access.

[49] Lei Y., Yang B., Jiang X., Jia F., Li N., Nandi A.K. (2020). Applications of machine learning to machine fault diagnosis: A review and roadmap. Mech. Syst. Signal Process..

[50] Chen T., Guestrin C. XGBoost: A scalable tree boosting system. Proceedings of the 22nd ACM SIGKDD International Conference on Knowledge Discovery and Data Mining.

[51] Wang X., Snášel V., Mirjalili S., Pan J.-S., Kong L., Shehadeh H.A. (2024). Artificial Protozoa Optimizer (APO): A novel bio-inspired metaheuristic algorithm for engineering optimization. Knowl.-Based Syst..

[52] Saito T., Rehmsmeier M. (2015). The precision-recall plot is more informative than the ROC plot when evaluating binary classifiers on imbalanced datasets. PLoS ONE.

